# Basilar Predominant Emphysema: Thinking beyond Alpha-1-Antitrypsin Deficiency

**DOI:** 10.1155/2022/9840085

**Published:** 2022-12-09

**Authors:** Ho-Man Yeung, Lauren Gaffaney

**Affiliations:** Department of Medicine, Lewis Katz School of Medicine, Temple University, Philadelphia, PA, USA

## Abstract

Basilar predominant emphysema, or disproportionate emphysematous involvement of the lung bases compared to the apices, is an uncommon radiographic pattern of emphysema traditionally associated with alpha-1-antitrypsin deficiency (AATD). We present a case of a 59-year-old female with 41 pack-year tobacco use, Stage IV COPD with supplemental oxygen, and bibasilar predominant emphysema who successfully underwent bronchoscopic lung volume reduction. She presented with recurrent hospitalizations for frequent exacerbations. After lung reduction, the patient displayed improvement in functional status without hospitalizations at the 15-month follow-up. Careful history taking is essential for any patients diagnosed with lower lobe emphysema to elucidate the underlying etiology. This case challenges the notion that basilar emphysema is sensitive or specific for AATD and emphasizes that this pattern of emphysema has a broad differential diagnosis and alternative etiologies should be considered. Our patient was ultimately diagnosed with smoking-related emphysema, with atypical bibasilar involvement. Furthermore, basilar predominant emphysema should be considered a separate entity from its apical predominant counterpart.

## 1. Introduction

Chronic obstructive pulmonary disease (COPD) is a common and severe disease involving airflow obstruction, of which one manifestation is emphysema. Alpha-1-antitrypsin deficiency (AATD) is an underrecognized genetic disorder involving the SERPINA1 gene, which causes Z-type alpha-1 antitrypsin molecules to form polymers [1]. Polymerization prevents secretion of the alpha-1-antitrypsin (AAT) molecules into the bloodstream, leading to the destruction of the lung parenchyma [1, 2]. This destruction predominantly affects the lower lobes, causing a bibasilar pattern of emphysema [2]. However, not all lower lobe emphysematous disease is due to AATD, and it is important to recognize that this pattern of emphysema can be seen in other etiologies including Swyer-James-MacLeod syndrome, occupational exposures, intravenous injection of crushed methylphenidate tablets, and tobacco use.

## 2. Case Presentation

**Figure 1:**
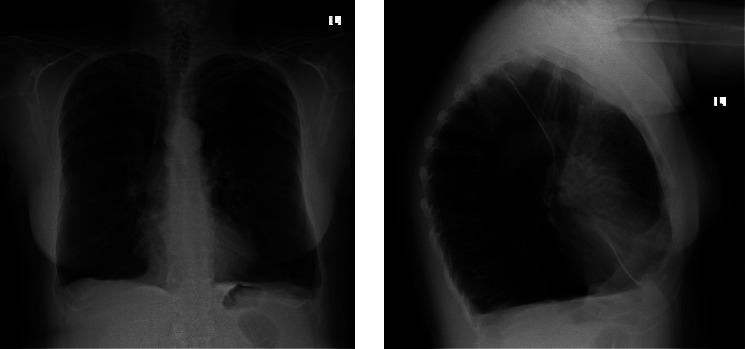
Chest X-rays of posterioanterior and lateral view showing bilateral lung hyperinflation, diaphragm flattening, and hyperlucency.

**Figure 2:**
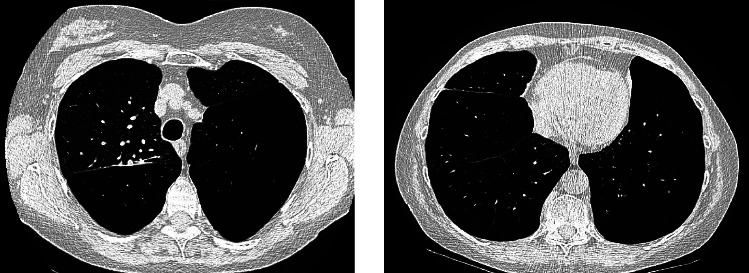
CT chest of upper and lower lobes showing panlobular emphysema with predominantly lower lobe involvement.

**Figure 3:**
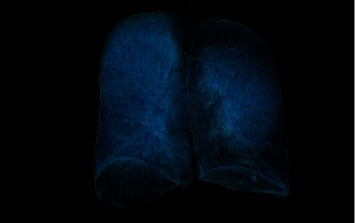
CT chest 3D reconstruction showing decreased lung field on bilaterally lower lobes and the left upper lobe.

A 59-year-old female with a history of cigarette smoking, obstructive sleep apnea, and Stage IV COPD on supplemental oxygen presented for evaluation of surgical management of her lung disease. Within the past year, she had five hospitalizations or emergency room visits for COPD exacerbations. She experienced increasing difficulty with physical activities despite using portable oxygen. She endorsed compliance with her medications, which include an albuterol inhaler and nebulized solution, fluticasone-umeclidinium-vilanter, omeprazole, and roflumilast. She previously worked at a chocolate factory but denied known exposure to fumes or chemicals. She was born and raised in the United States and denied any recent travel. Additionally, she denied any history of childhood pneumonia or respiratory problems. Family history was unremarkable. She quit smoking 15 months prior to the presentation but had a total 41 pack-year history. She denied using vaporized tobacco. She endorsed occasional drinking and denied any illicit drug use, including injection drug use. AAT level was 169 mg/dL and genotype PI *∗* MM, which were inconsistent with a diagnosis of AATD. Pulmonary function testing (PFT) demonstrated an obstructive pattern with FEV1 0.71L (28%), FVC 1.36L (38%), FEV1/FVC ratio 0.56, TLC 7.9L (150% of predicted), and DLCO 26%. Right heart catheterization showed mean pulmonary arterial pressure of 25 mmHg and pulmonary vascular resistance 3.76 Woods units. Chest X-rays showed bilateral lung hyperinflation, diaphragm flattening, and hyperlucency (Figure 1), and CT chest showed panlobular emphysema with predominantly lower lobe involvement (Figure 2). A 3D reconstruction of the CT showed decreased lung fields on bilaterally lower lobes and the left upper lobe (Figure 3). Due to her progressive symptoms, she underwent bronchoscopic lung volume reduction of the lower lobes. The left lower lobe was intervened first, with the placement of 8 Zephyr valves, followed by her right lower lobe two months later with 6 Zephyr valves placed. She tolerated both procedures well, without complication, and was promptly discharged home. In the 15 months following her BLVR, she has not had any urgent care visits or hospitalizations, requires no supplemental oxygen, and reports increased exercise tolerance. Repeat PFT showed improvements including FEV1 1.07L (40%), FVC 1.77L (60%), FEV1/FVC ratio 0.56, TLC 6.1L (115% of predicted), and DLCO 30%.

## 3. Discussion

The distribution of emphysema in a patient can be helpful in determining the underlying cause of the emphysema and has their own distinct differential diagnoses. Lower lobe disease is distinct from its upper lobe counterpart in severity of impairment [3], physiology [4], and increased mortality with LVRS [5]. In this patient, the differential diagnosis for lower lobe emphysema is broad and can be distinguished by careful history-taking, including smoking/drug use, occupational exposure, family history, and childhood infections. Here, we discuss each diagnosis considered.

AATD is an underrecognized disease with pulmonary and hepatic manifestations. The proposed mechanism for emphysema is the imbalance between a serine protease and its inhibitor leading to parenchymal destruction. Cigarette smoking and recurrent infections often accelerate AATD progression [2]. AATD should be suspected in patients with emphysema diagnosed at a young age (<45 years old), nonsmokers, a family history of early lung disease, or basilar predominant distribution. Diagnosis is confirmed by low AATs followed by genotyping showing homozygous Z alleles [2]. One rare genotype of AATD is the FF genotype. The F allele produces a normal quantity yet dysfunctional AAT, and thus, the risk of pulmonary manifestation is similar to that of ZZ AATD. Simultaneous testing of both the AAT level and genotype is preferred. The patient's AAT level and genotype were determined to be inconsistent with AATD.

Intravenous drug use of crushed talc- or magnesium silicate-containing tablets such as methylphenidate causes perivascular deposition leading to chronic inflammation [6]. It is linked to pulmonary hypertension and emphysema, particularly in the lower lobes [7]. However, our patient displayed no drug use behaviors.

Occupational exposure to fumes and chemicals is well known to cause a large spectrum of lung disease. Chronic exposure to diacetyl, a volatile organic compound that is used as flavoring chemical, causes airway epithelial injury which leads to bronchiolitis obliterans, called “popcorn lung” [8]. Bronchiolitis obliterans causes fibrosis due to excessive granulation tissue affecting the small conducting airways and can mimic COPD on imaging but often results in a restrictive or mixed restrictive/obstructive pattern on pulmonary function testing [9]. “Popcorn lung” was considered given her occupational history; however, her PFTs were not consistent with the restrictive disease.

Swyer-James-MacLeod syndrome is a rare entity associated with postinfectious bronchiolitis obliterans, typically during childhood [10]. The characteristic radiological pattern is hypoplasia/hypogenesis of the pulmonary arteries with hypoperfusion, giving a translucency to unilateral lung. It is associated with recurrent pulmonary infections during childhood [10]. The patient in our case underwent CTA of the chest which did not reveal any hypogenesis of the pulmonary arteries. Furthermore, she denied recurrent childhood respiratory infections.

Cigarette smoking remains the most important risk factor for developing emphysema [11]. Smoking-related emphysema is typically upper lobe predominant [12], although lower lobe predominant emphysema can be seen [13]. This patient's significant smoking history and negative workup for other causes made smoking-related emphysema most likely.

This patient had Stage IV COPD with frequent exacerbations despite maximum medical therapy. The bibasilar manifestation of emphysema is most likely smoking-related. Smoking-related emphysema is typically upper lobe predominant, and bibasilar manifestation is relatively uncommon. She was offered lung volume reduction at our lung center, with clinical improvement in her symptoms and functional status. Although LVRS is the current gold standard treatment of hyperinflation in COPD with mortality and symptomatic benefits, it also confers an increased risk of perioperative morbidity. These benefits are most obvious in patients with upper lobe predominant disease and poor functional status [14]. On the contrary, patients with nonupper lobe predominant emphysema are likely poor candidates for LVRS. BLVR is a minimally invasive technique for lung volume reduction using either endobronchial valves or coils. It has been shown to offer improvement in exercise tolerance and lung function for patients who do not qualify for LVRS or are poor surgical candidates. Similar to LVRS, greater benefits are seen in patients with upper lobe predominant emphysema. To date, no large studies have shown improved survival with BLVR, but it still remains an option for patients with severe COPD for symptomatic management [15].

## 4. Conclusion

While lower lobe emphysema is commonly attributed to AATD, the differential diagnosis is broad and often distinct from its upper lobe counterpart. Careful history taking is essential for any patients diagnosed with lower lobe emphysema to elucidate the underlying etiology.

## Abbreviations

COPD: Chronic obstructive pulmonary disease
AATD: Alpha-1-antitrypsin deficiency
AAT: Alpha-1-antitrypsin
OSA: Obstructive sleep apnea
FEV1: Forced expiratory volume per 1 second
FCV: Forced vital capacity
TLC: Total lung capacity
RV: Residual volume
DLCO: Diffusing capacity for carbon monoxide
LVRS: Lung volume reduction surgery
BLVR: Bronchoscopic lung volume reduction.
